# Elements of Materia Medica and Pharmacy

**Published:** 1886-06

**Authors:** 


*Elements of Materia Medica and Pharmacy.* By ALFRED W. GERRARD, Examiner to the Pharmaceutical Society, &c., &c. London: H. K. LEWIS, 136 Gower Street. 1886.

An excellent book, which fulfils its pretensions. It is scarcely a work which is of value to the practitioner, but appeals to the medical student in the earlier part of his career. Mr. Gerrard's experience at University College Hospital has taught him what the examining bodies expect the student to know of Pharmacy and Materia Medica, and he has prepared a well-arranged manual to help to the attainment of that knowledge. The book partakes rather of the nature of a

"cram," but the acquirement of an ephemeral knowledge of Pharmacy is always more or less of that kind. It is clearly printed on good paper, and free from mistakes and clerical errors, so far as we have observed; and to students about to go up to the examination in Materia Medica of the Conjoint Board is to be strongly recommended, the slight amount of therapeutical information given being sufficient for that purpose.

